# Data on thermal conductivity and dynamic mechanical properties of graphene quantum dots in epoxy

**DOI:** 10.1016/j.dib.2019.105008

**Published:** 2019-12-21

**Authors:** Joel R. Seibert, Ozgur Keles, Jun Wang, Folarin Erogbogbo

**Affiliations:** a Department of Biomedical, Chemical, and Materials Engineering, San Jose State University, 1 Washington Square, San Jose, CA, 95112, United States; b Thermal and Mechanical Characterization Lab, PerkinElmer, 75 Nicholson Ln, San Jose, CA, 95134, USA

**Keywords:** Graphene quantum dots, Thermal conductivity, Dynamic mechanical analysis, Graphene polymers, Differential scanning calorimetry, Graphene nanoparticles, Graphene polymers

## Abstract

Graphene Quantum Dots (GQDs) and epoxy have been combined into a nanocomposite and evaluated for their thermal and dynamic mechanical properties. Samples of varying GQD mass loading were first examined with SEM in several images. Thermal conductivity was estimated using Differential Scanning Calorimetry (DSC) with a step analysis technique and analysis program. Several dynamic mechanical properties were recorded using Dynamic Mechanical Analysis (DMA) and displayed in their raw and analyzed formats. For more insight please see Infusion of graphene quantum dots to modulate thermal conductivity and dynamic mechanical properties of polymers [1].

**Table:** Specifications Table

Subject area	Materials Science
More specific subject area	Graphene nanocomposites
Type of data	Tables and plots
How data was acquired	FEI XL30 ESEM TMP Perkin Elmer DMA-8000 Perkin Elmer DSC-8500
Data format	Raw and Analyzed
Experimental factors	Dispersement, degassing, curing temperature and time.
Experimental features	Nanocomposite fabrication, SEM, TEM, DSC, DMA
Data source location	Thermal and Mechanical Characterization Lab, PerkinElmer, 75 Nicholson Ln, San Jose, CA 95134
Data accessibility	https://figshare.com/s/db2e99cbb17ec8fc5481 https://drive.google.com/open?id=1w4pX7BrSwf5zrJxIoItv1Vrk3fxBssgf
Related research article	Author's name: Seibert JR, Keleş Ö, Wang J, Erogbogbo F. Title: Infusion of graphene quantum dots to modulate thermal conductivity and dynamic mechanical properties of polymers Journal: Polymer. 2019; 121988. https://doi.org/10.1016/j.polymer.2019.121988

**Table:** 

**Value of the Data** • Shows improved properties with addition of Graphene Quantum Dots (GQDs) in a nanocomposite when compared to other graphemic nanocomposites. • Validates method of estimating thermal conductivity using DSC. • Provides insight to the thermal and mechanical mechanisms of GQD/Epoxy composite properties.

## 1 Data

The dataset contains raw files related to the manuscript, “Infusion of graphene quantum dots to modulate thermal conductivity and dynamic mechanical properties of polymers [1]”. The raw data obtained from for scanning electron microscopy was used in the construction, Differential scanning calorimetry, thermal conductivity, and dynamic mechanical analysis.

### 1.1 SEM

SEM Images are found in the linked data repository in the SEM folder for E-GQD samples of with GQD mass loading of 0%, 1%, 2.5% and 5%. Images were taken with magnifications ranging from 100x-10000x.

### 1.2 DSC

Thermal conductivity data analyzed by a program received from the University of Rostockthat was used in research by Merzlyakov et al. is found in the linked data repository in the file TC.Combined.xlsx [2].

DSC was also used to verify the cure of the epoxy in samples, data related to this verification in found in the linked data repository in the file named epoxycurecombined.xlsx.

### 1.3 DMA

Raw and analyzed data for DMA tests on samples with GQD mass loading of 0%, 1%, 2.5% and 5% are found on the linked data repository in the file named combined.DMA.xlsx.

## 2 Experimental design, materials, and methods

### 2.1 TEM

TEM images were obtained using Hitachi H-9500 System at 300 kV in a bright field mode at NASA Ames. Drops of GQD solution were on lacey carbon 300 mesh grids, which then were dried at room temperature for 20 minutes to remove any liquid from the GQDs. Images taken with the TEM were used to verify the structure of GQDs and compare to the bird charcoal source material.

### 2.2 SEM

Fractured GQD/Epoxy composite samples were examined under SEM using the FEI XL30 ESEM TMP at NASA Ames. The SEM was used in low vacuum water mode, with a 15 kV beam. GQD/Epoxy samples of 1%, 2.5%, 5% and neat epoxy were made using the DMA procedure but were fractured using a hammer and a vice, the fracture surface was examined in the SEM.

### 2.3 DSC

Thermal conductivity was measured using DSC with the method previously mentioned research by Merzlyakov et al. in a program received from the University of Rostock available at http://www.polymerphysik.uni-rostock.de/research/research.php. Measuring thermal conductivity using DSC requires additional sample preparation and a modulated temperature scan. This technique is considered an estimation which is within 5% of the actual thermal conductivity. A Perkin Elmer DSC-8500 dual furnace DSC was used for this experiment. A temperature step profile was used with a starting temperature of 40 C with 1 C heating steps at a rate of 75 C/min, with 1 min isothermic holds with 5 repetitions steps to a final temperature of 45 C. First, the temperature profile was run and measured with an empty furnace and reference furnace to establish a baseline. Then the profile was run with two different sapphire standards one of which has a mass at least double the other standard as specified in the manual for the software obtained from University of Rostock.

Samples were cast into DSC pans with diameter approximately 6.5 mm and then sanded and polished to approximately 0.50 mm with 600 grit sandpaper. After samples had been polished, a small amount of high temperature silicone Apiezon H grease was applied for good thermal contact with the DSC. Once samples had been measured with the step profile, the program from University of Rostock was used to calculate the thermal conductivity and heat capacity using the method described in the literature review of Merzlyakov et al.’s research.

### 2.4 DMA

Dynamic Mechanical Analysis was performed at PerkinElmer in 3-point bend configuration using the PerkinElmer DMA-8000. Samples for DMA were cast into the previously mentioned mold. Samples were carefully removed from the mold. Samples for DMA were tested in the 3-point bend configuration in a PerkinElmer DMA 8000. Samples were tested at 1 cycle per second and a strain of .0050 mm. Analysis of the mechanical response measured by DMA using PerkinElmer software gave a calculated storage modulus and glass transition temperature. Glass transition temperature for DSC and DMA differ in that DSC measures when the endothermic or exothermic phase change occurs, whereas DMA measures when the solid behaves like a glass.
